# A New Approach to Unwanted-Object Detection in GNSS/LiDAR-Based Navigation

**DOI:** 10.3390/s18082740

**Published:** 2018-08-20

**Authors:** Mathieu Joerger, Guillermo Duenas Arana, Matthew Spenko, Boris Pervan

**Affiliations:** 1 College of Aerospace & Mechanical Engineering, The University of Arizona, Tucson, AZ 85721, USA; 2 Armour College of Engineering, Illinois Institute of Technology, Chicago, IL 60616, USA; gdueasar@hawk.iit.edu (G.D.A.); mspenko@iit.edu (M.S.); pervan@iit.edu (B.P.)

**Keywords:** navigation, safety, GNSS, LiDAR, detection, integrity monitoring, autonomous cars

## Abstract

In this paper, we develop new methods to assess safety risks of an integrated GNSS/LiDAR navigation system for highly automated vehicle (HAV) applications. LiDAR navigation requires feature extraction (FE) and data association (DA). In prior work, we established an FE and DA risk prediction algorithm assuming that the set of extracted features matched the set of mapped landmarks. This paper addresses these limiting assumptions by incorporating a Kalman filter innovation-based test to detect unwanted object (UO). UO include unmapped, moving, and wrongly excluded landmarks. An integrity risk bound is derived to account for the risk of not detecting UO. Direct simulations and preliminary testing help quantify the impact on integrity and continuity of UO monitoring in an example GNSS/LiDAR implementation.

## 1. Introduction

This paper describes the design, analysis, and preliminary testing of a new method to quantify safety in GNSS/LiDAR navigation systems. An integrity risk bound is derived, which accounts for failures to detect undesirable, unmapped and wrongly extracted obstacles. The paper describes an innovation-based method, which is an alternative to the solution separation approach used in [1]. In addition, the paper provides the means to quantify the impact of unwanted objects (UO) on the risk of incorrect association. This work is intended for driverless cars, or highly automated vehicles (HAV) [2,3], operating in changing environments where unknown, moving obstacles (cars, buses, and trucks) are not wanted as landmarks for localization, and may occlude other useful, mapped landmarks. 

This research leverages prior analytical work carried out in civilian aviation navigation where safety is assessed in terms of integrity and continuity [4]. These performance metrics are sensor- and platform-independent. Integrity is a measure of trust in sensor information: integrity risk is the probability of undetected sensor errors causing unacceptably large positioning uncertainty [4]. Continuity is a measure of the navigation system’s ability to operate without unscheduled interruption. Both loss of integrity and loss of continuity can place the HAV in hazardous situations [4,5].

Several methods have been established to predict integrity and continuity risks in GNSS-based aviation applications [6,7,8]. Unfortunately, the same methods do not directly apply to HAVs, because ground vehicles operate under sky-obstructed areas where GNSS signals can be altered or blocked by buildings and trees.

HAVs require sensors in addition to GNSS, including LiDARs, cameras, or radars. This paper focuses on LiDARs because of their prevalence in HAVs, of their market availability, and of our prior experience. A raw LiDAR scan is made of thousands of data points, each of which individually does not carry any useful navigation information. Raw measurements must be pre-processed before they can be used to estimate HAV positioning and orientation (or pose). 

A first class of algorithms establishes correlations between successive scans to estimate sensor changes in ‘pose’ (i.e., position and orientation) [9,10,11,12]. These procedures, including the Iterative Closest Point (ICP) approach [13], can become cumbersome when evaluating safety of HAVs moving over time. A second class of algorithms provides sensor localization by tracking recognizable, static features in the perceived environment (seminal references and survey papers can be found in [14,15,16,17,18,19]). Features can include, for example, lines or planes corresponding to building walls in two- or three-dimensional scans, respectively. Previous knowledge of feature parameters can be provided either from a landmark map, or from past-time estimation in Simultaneous Localization and Mapping (SLAM) [15,20]. The resulting information can then be iteratively processed using sequential estimators in SLAM (e.g., Extended Kalman filter or EKF), which is convenient in practical implementations. To estimate the HAV’s pose starting from a raw LiDAR scan, two intermediary, pre-estimator procedures must be carried out: feature extraction (FE), and data association (DA). 

First, FE aims at finding the few most consistently recognizable, viewpoint-invariant, and mutually distinguishable landmarks in the raw sensor data. Second, DA aims at assigning the extracted features to the corresponding feature parameters assumed in the estimation process, i.e., at finding the ordering of mapped landmarks that matches the ordering of extracted features over successive observations. Incorrect association is a well-known problem that can lead to large navigation errors [21], thereby representing a threat to navigation integrity. FE and DA can be challenging in the presence of sensor uncertainty. This is why many sophisticated algorithms have been devised [17,18,19,21,22,23]. But, *how can we prove whether FE and DA are safe for life-critical HAV navigation applications*?

This research question is mostly unexplored. Several publications on multi-target tracking describe relevant approaches to evaluate the probability of correct association in the presence of measurement uncertainty [24,25]. However, these algorithms are not well suited for safety-critical HAV applications due to their lack of prediction capability, to approximations that do not necessarily upper-bound risks, and to high computational loads. Also, the risk of FE is not addressed. Overall, research on integrity and continuity of FE and DA is sparse. 

This paper builds upon prior work in [1,26,27,28], where we developed an analytical integrity risk prediction method using a multiple-hypothesis innovation-based DA process. We established a compact expression for the integrity risk of LiDAR-based pose estimation over successive iterations. However, references [26,27,28] made simplifying assumptions that limit the applicability of these prior results. For example, we assumed that the set of landmarks in the a-priori map was exactly the same as the one being extracted. This assumption was relaxed in [1] where we developed an integrity-risk-minimizing data-selection method. To achieve this, we derived a bound on the risk of incorrect association, with which a subset of measurements can be used while considering potential wrong associations with all landmarks surrounding the LiDAR. This bound was used in a preliminary approach to detect UO using solution separation tests. In practice, UO such as other vehicles passing by are likely to be extracted, and may even occlude other mapped landmarks. Obstacle detection methods have been developed to mitigate the impact of such UOs (example methods are described in [29,30]). But, the safety risks of using UOs as landmarks for navigation have yet to be fully quantified.

In response, in this paper, we derive new methods to quantify the integrity risk caused by failures to detect unwanted obstacles (UO), while guaranteeing a predefined false alert risk requirement. 

Section 2 of the paper provides an overview of the risk evaluation methods developed in [1,26,27,28], and of their limitations. These methods use a nearest-neighbor DA criterion [9], defined by the minimum normalized norm of the EKF innovation vectors over all possible landmark permutations. Section 3 and Section 4 deal with the situation where a mapped landmark is not extracted, but another unknown obstacle is extracted instead (e.g., case of an obstacle masking a mapped landmark). This paper assumes that UOs only mask one unknown landmark at a time as the HAV drives by. Section 3 describes the innovation-based approach employed to detect the UO (which differs from the solution separation detector employed in [1]). An integrity risk bound is then derived to incorporate the risk of not detecting a UO when one might be present. This bound is analytically evaluated in two steps in Section 4: we account for the impact of undetected UO: (a) on the probability of hazardously misleading information (HMI) under the correct association (CA) hypothesis, and (b) on the probability of incorrect association (IA). Navigation integrity performance is then assessed in Section 5 using direct simulations and preliminary testing for an example implementation using GNSS and two-dimensional LiDAR data.

## 2. Background: Integrity Risk Bound Accounting for Incorrect Associations

This section presents an overview of the integrity risk evaluation method described in [1,26,28], which uses a multiple-hypothesis innovation-based DA process. 

### 2.1. Integrity Risk Definition and Integrity Risk Bound 

**Figure 1:**
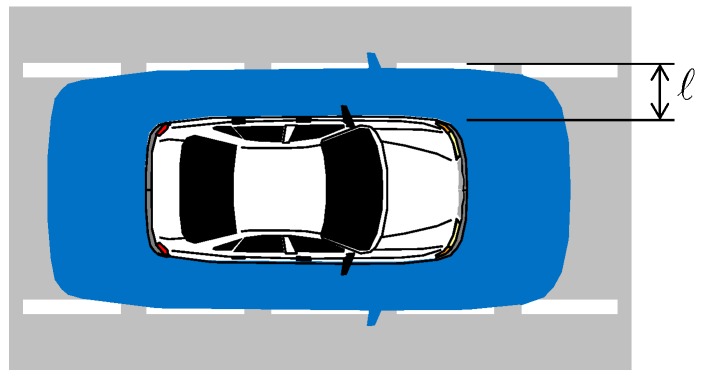
Defining Integrity Risk for Automotive Applications. The integrity risk is the probability of the car being outside the alert limit requirement box (blue shaded area) when it was estimated to be inside the box. When lateral deviation is of primary concern, then the alert limit is the distance ℓ between edge of car and edge of lane.

The integrity risk, or probability of hazardous misleading information (HMI) at time k, is noted $P(HMI_{k})$, and is defined in Figure 1. The safety criterion is: $P(HMI_{k})\leq I_{REQ,k}$ where $I_{REQ,k}$ is a predefined integrity risk requirement set by a certification authority (similar to requirements set for aviation applications in [4,8]). Values for $I_{REQ,k}$ that might be used in future HAV applications can be found in [5].

In [26,28], we established an analytical bound on the integrity risk, which accounts for the risk of incorrect associations. This bound is expressed as:

(1) $$P(HMI_{k})\leq 1-\left[1-P(HMI_{k}|CA_{K})\right]P(CA_{K})+I_{FE,k}$$

with

(2) $$P(HMI_{k}|CA_{K})=2Q\left\{\ell /\sigma_{k}\right\}$$

(3) $$P(CA_{K})\geq \prod_{l=1}^{k}P_{\chi^{2}}\left\{n_{l}+m_{l},\frac{L_{l}^{2}\lambda_{l}^{2}}{4}\right\}$$

where
kis an index identifying a time step;Kdesignates a range of indices: K≡{0,…,k}, from filter initiation to time k;$CA_{K}$is the correct association hypothesis for all landmarks, at all times 0, ..., k;Q{ }is the tail probability function of the standard normal distribution;ℓis the specified alert limit that defines a hazardous situation [4,5,8] (e.g., see Figure 1);$\sigma_{k}$is the standard deviation of the estimation error for the vehicle state of interest (or linear combination of states);$P_{\chi^{2}}\{dof,T\}$is the probability that a chi-squared-distributed random variable with “*dof*” degrees of freedom is lower than some value *T*;$n_{l}$is the number of measurements at time step l;$m_{l}$is the number of estimated state parameters at time step l;$I_{FE,l}$is an integrity risk budget allocation, i.e., a fraction of $I_{REQ,k}$ that we choose to satisfy: $I_{FE,k}<<I_{REQ,k}$;$L_{l}^{2}$is the minimum mean normalized separation between landmark features that can be guaranteed with probability larger than $1-I_{FE,l}$. The normalized feature separation metric is derived in [28]. $L_{l}^{2}$ is derived at FE using a map or database of landmarks or using landmark observations at previous time-steps in SLAM;$\lambda_{l}^{2}$is a mapping coefficient from separation space to EKF innovation space. This coefficient is determined by solving an eigenvalue problem in [28]. The minimum eigenvalue is taken to lower bound $P(CA_{K})$, which is conservative;$L_{l}^{2}\lambda_{l}^{2}$forms a probabilistic lower bound on the mean innovation’s norm, which is further described in the Section 2.2.

The integrity risk bound in Equation (1) is refined in this paper to account for the presence of UOs and for failures to detect them. Equation (1) captures a key tradeoff in data association: on the one hand, using only few measurements can cause a large nominal estimation error and hence large $P(HMI_{k}|CA_{K})$; but on the other hand, few measurements from sparsely distributed landmarks can improve $P(CA_{K})$ because features are “separated”, distinguishable, and therefore can be robustly associated. $P(HMI_{k})$ is unknown, but we can assess safety by comparing $I_{REQ,k}$ to the upper bound given in Equations (1)–(3), where all terms are known. 

### 2.2. Innovation-Based Data Association

Equation (1) is derived for an innovation-based DA process, which is further described in the following paragraphs. Let $n_{L}$ be the total number of visible landmarks and $n_{F}$ the number of estimated feature parameters per landmark. Feature parameters can include landmark position, size, orientation, surface properties, etc. When using LiDAR only (we integrate GNSS in Section 5), the total number of feature parameters within the visible landmark set is: $n_{k}\equiv n_{L}n_{F}$. We can stack the actual (true) values of the extracted feature parameters for all landmarks in an $n_{k}\times 1$ vector $z_{k}$. Let ${\hat{z}}_{k}$ be an estimate of $z_{k}$. We assume that the cumulative distribution function of ${\hat{z}}_{k}$ can be bounded by a Gaussian function with mean $z_{k}$ and covariance matrix $V_{k}$ [31,32,33]. We use the notation: ${\hat{z}}_{k}\sim N(z_{k},V_{k})$. 

The nonlinear measurement equation can be written in terms of the $m_{k}\times 1$ state parameter vector $x_{k}$ as

(4) $${\hat{z}}_{k}=h_{k}(x_{k})+v_{k}$$

where
$x_{k}$includes vehicle pose parameters and may also include landmark feature parameters (for SLAM-type approaches);$v_{k}$is the extracted measurement noise vector: $v_{k}\sim N(0_{n\times 1},V_{k})$, where $0_{a\times b}$ is an a×b matrix of zeros.

The mean of ${\hat{z}}_{k}$ is $z_{k}=h_{k}(x_{k})$. Equation (4) can be linearized about an estimate ${\bar{x}}_{k}$ of $x_{k}$:

(5) $${\hat{z}}_{k}\approx h_{k}({\bar{x}}_{k})+H_{k}(x_{k}-{\bar{x}}_{k})+v_{k}\;\mathrm{where}\;H_{k}\equiv {\frac{\partial h_{k}(x_{k})}{\partial x_{k}}|}_{{\bar{x}}_{k}}$$

The ordering of landmarks in ${\hat{z}}_{k}$ is arbitrary and unknown. A nearest-neighbor approach (described below) is used to determine the ordering of measurement-to-state coefficients in $h_{k}({\bar{x}}_{k})$ and $H_{k}$. Failing to find the landmark ordering that matches that of ${\hat{z}}_{k}$ causes estimation errors called incorrect associations (IA).

If $n_{L}$ landmarks are extracted, there are $\left(n_{L}!\right)$ ways to arrange measurements in ${\hat{z}}_{k}$, which we call $\left(n_{L}!\right)$ candidate associations. For clarity of exposition, we assume that the total number of mapped landmarks, or of previously observed landmarks when using SLAM, is also the number $n_{L}$ of extracted landmarks (procedures to address this assumption are given in [1]). Let subscript i designates association hypotheses, for $i=0,\ldots ,n_{A}$, where $n_{A}=n_{L}!-1$. We define i=0 the fault-free, correct association (CA) hypothesis, and the other $n_{A}$ hypotheses are IA. IA impacts the EKF estimation process through the innovation vector $\gamma_{i,k}$. Vector $\gamma_{i,k}$ is an effective indicator of CA because it is zero mean only for the correct association. 

In all IA cases, the mean of $\gamma_{i,k}$ is not zero and is expressed in terms of n×n permutation matrices $A_{i,k}$, for $i=1,\ldots ,n_{A}$, as

(6) $$\begin{array}{cc} \gamma_{i,k} & ={\hat{z}}_{k}-A_{i,k}h_{k}({\bar{x}}_{k})\\ & =y_{i,k}+v_{k}-A_{i,k}H_{k}{\bar{\varepsilon }}_{k} \end{array}$$

where

(7) $$y_{i,k}\equiv h_{k}(x_{k})-A_{i,k}h_{k}(x_{k})=(I_{n}-A_{i,k})z_{k}\;\mathrm{and}\;y_{0,k}=0$$

where ${\bar{\varepsilon }}_{k}$ is the EKF state prediction error vector (${\bar{\varepsilon }}_{k}\equiv {\bar{x}}_{k}-x_{k}$) and $I_{a}$ is the a×a identity matrix.

Let ${\bar{P}}_{k}$ be the EKF state prediction error covariance matrix. We select the association candidate that satisfies the nearest-neighbor association criterion [9], defined as

(8) $$\underset{i=0,\ldots ,n_{A}}{\min}\gamma_{i,k}^{2}$$

where

(9) $$\gamma_{i,k}^{2}\equiv \gamma_{i,k}^{T}Y_{i,l}^{-1}\gamma_{i,k}\;\mathrm{and}\;Y_{i,k}=A_{i,k}H_{k}{\bar{P}}_{k}H_{k}^{T}A_{i,k}^{T}+V_{k}\;\mathrm{for}\;i=0,\ldots ,n_{A}$$

The probability of correct association is the probability of the following event occurring: $\overset{n_{A}}{\underset{i=1}{\cap }}\left\{\gamma_{0,k}^{2}\leq \gamma_{i,k}^{2}\right\}$. We can determine the a priori distributions of variables $\gamma_{i,k}^{2}$, for $i=0,\ldots ,n_{A}$, except their mean values that are unknown. In [28], we show that the term $L_{l}^{2}\lambda_{l}^{2}$ used in Equation (1) is a lower bound on the mean innovation’s norm $y_{i,l}^{2}$ ($y_{i,l}^{2}\equiv y_{i,l}^{T}Y_{i,l}^{-1}y_{i,l}$). Equation (1) is a bound on $P(HMI_{k})$, but it assumes that no UO is present. We first design a UO detector and derive a new $P(HMI_{k})$ bound in Section 3, and then we establish an analytical method to evaluate the impact of undetected UOs on this new bound in Section 4.

## 3. Risks Involved with Unwanted Object Detection 

In the presence of a UO, the innovation vector’s norm in Equation (9) is nonzero under all association hypotheses. In this case, the correct association hypothesis must be redefined. We call correct association (CA) the one where all landmarks that are not occluded by a UO are correctly associated, i.e., where the innovation vector would be zero mean if the UO was removed. The nonzero mean in the CA’s innovation vector is caused by the UO only, not by other incorrectly associated landmarks.

### 3.1. Innovation-Based Detector

If a UO is present, $\gamma_{i,k}$ does not have a mean of zero even under CA. To identify such events, we can set a threshold $T_{k}^{2}$ on the minimum innovation norm squared, or, since the process is performed over time, on the running sum of minimum innovation norms squared. Using innovations (instead of solution separations as in [1]) will facilitate evaluation of $P(CA_{K})$ in Section 4. The UO detection test statistic is defined as

(10) $$q_{k}^{2}=\sum_{l=0}^{k}\underset{i=0,\ldots ,n_{A}}{\min}\gamma_{i,l}^{2}$$

Since the innovation sequence is white, $q_{k}^{2}$ is non-centrally chi-squared distributed with $n_{DOF,k}=\sum_{l=0}^{k}n_{l}$ degrees of freedom and noncentrality parameter (NCP) $\mu_{Q,k}^{2}$. We use the notation $q_{k}^{2}\sim \chi^{2}(n_{DOF,k},\mu_{Q,k}^{2})$. $\mu_{Q,k}^{2}$, which is further discussed in Section 4. The detection threshold $T_{k}^{2}$ is set according to a continuity risk requirement $C_{REQ}$ to limit the risk of false alerts. False alerts occur when no UO is present, causing $q_{k}^{2}$’s NCP to be zero under CA. Thus, $T_{k}^{2}$ is given by

(11) $$\int_{0}^{T_{k}^{2}}\chi_{\tau }^{2}\left(n_{DOF,k},0\right)d\tau =1-C_{REQ}\;\mathrm{or}\;\mathrm{equivalently},T_{k}^{2}=P_{\chi^{2}}^{-1}\left\{n_{DOF,k},1-C_{REQ}\right\}$$

where $P_{\chi^{2}}^{-1}\left\{\;\right\}$ is the inverse cumulative distribution function (CDF) of the chi-squared distribution $\chi_{\tau }^{2}(n_{DOF,k},0)$ evaluated at the $1-C_{REQ}$ quantile.

If $T_{k}^{2}$ is exceeded, we interrupt the mission. (As an alternative to mission interruption, we could select a different set of landmark feature measurements as in [1,34], but this is beyond the scope of this paper.) This does not impact $P(HMI_{k})$. However, if $T_{k}^{2}$ is not exceeded, a UO may still be present because the detection test statistic $q_{k}^{2}$ is a random, noisy variable. Navigation errors due to undetected UOs can cause the vehicle to crash.

### 3.2. Integrity Risk in Presence of UO

To quantify the integrity risk caused by potentially undetected UOs, the $P(HMI_{k})$ definition in Equation (1) is modified: HMI is the joint event of the car being out of lane while no alert has been sent. The integrity risk is redefined as

(12) $$P(HMI_{k})=P\left(|{\hat{\varepsilon }}_{k}|>\ell \cap \left[\overset{k}{\underset{l=0}{\cap }}q_{l}^{2}\leq T_{l}^{2}\right]\right)$$

where ${\hat{\varepsilon }}_{k}$ is the EKF state estimation error for the state of interest, e.g., for the vehicle’s lateral deviation within its lane. Because ${\hat{\varepsilon }}_{k}$ and $q_{k}^{2}$ are obtained after associating LiDAR data to a landmark map, we consider a set of mutually exclusive, exhaustive hypotheses of correct associations (CA) and incorrect associations (IA). We derived the following bounds:

(13) $$\begin{array}{l} P(HMI_{k})\leq P(HI_{k}\cap ND_{K}\cap CA_{K})+P(HI_{k}\cap ND_{K}\cap IA_{K})+I_{FE,k}\\ \;\leq P(HI_{k}\cap ND_{k}|CA_{K})+P(ND_{K}\cap IA_{K})+I_{FE,k} \end{array}$$

where
$HI_{k}$is the event of hazardous information (HI) at time k, defined as $HI_{k}\equiv |{\hat{\varepsilon }}_{k}|>\ell$;$ND_{K}$is the event of no detection (ND) at all previous times 0, ..., k, defined as $ND_{K}\equiv \overset{k}{\underset{l=0}{\cap }}q_{l}^{2}\leq T_{l}^{2}$;$ND_{K}$is the event of ND at time k, defined as $ND_{K}\equiv q_{k}^{2}\leq T_{k}^{2}$;$CA_{K}$is the CA hypothesis for all landmarks, at all times 0, ..., k;$IA_{K}$is the IA hypothesis for any landmarks, at any time 0, ..., k.

In Section 4, we derive upper bounds on $P(HI_{k}\cap ND_{K}|CA_{K})$ and $P(ND_{K}\cap IA_{K})$.

## 4. Analytical Bounds on Risks Caused by Undetected Unwanted Objects

As stated in Section 1, this paper assumes that UOs only mask one unknown landmark at a time as the HAV drives by. This can be extended to multiple UOs masking one subset of landmarks at a time, using the procedures described in [1]. However, the performance analysis in Section 5 does not illustrate this case. The limitation is that the UO-free subset must be large enough to enable HAV pose estimation; the method requires landmark redundancy because it assumes an uncertain vehicle dynamic model and no inertial navigation system. 

### 4.1. Risk of HMI Due to Undetected UO

We consider a set of mutually exclusive, exhaustive hypotheses $H_{h}$ of a UO masking a landmark h (or landmark subset h) for $h=0,\ldots ,n_{H}$, where $n_{H}$ is the total number of hypotheses. We note $H_{0}$ the fault-free (no UO) hypothesis. Using the law of total probability, $P(HI_{k}\cap ND_{K}|CA_{K})$ is rewritten as

(14) $$P(HI_{k}\cap ND_{k}|CA_{K})=\sum_{h=0}^{n_{H}}P\left(HI_{k}\cap ND_{k}\cap H_{h}|CA_{K}\right)$$

We have no prior knowledge on the probability of occurrence of $H_{h}$, but we can bound the sum of their occurrence probabilities by 1. Thus, $P(HI_{k}\cap ND_{K}|CA_{K})$ can be upper-bounded using the following expression:

(15) $$P(HI_{k}\cap ND_{k}|CA_{K})\leq \underset{h=0,\ldots ,n_{H}}{\max}P\left(|{\hat{\varepsilon }}_{k}|>\ell \cap q_{k}^{2}\leq T_{k}^{2}|H_{h}\cap CA_{K}\right)$$

Recalling that ${\hat{\varepsilon }}_{k}$ and $q_{k}^{2}$ are statistically independent (e.g., [35,36]), we can rewrite the bound in Equation (15) as

(16) $$P(HI_{k}\cap ND_{k}|CA_{K})\leq \underset{h=0,\ldots ,n_{H}}{\max}P(|{\hat{\varepsilon }}_{k}|>\ell |H_{h}\cap CA_{K})P(q_{k}^{2}\leq T_{k}^{2}|H_{h}\cap CA_{K})$$

Under the correct association hypothesis ($CA_{K}$), the distributions of ${\hat{\varepsilon }}_{k}$ and $q_{k}^{2}$ are known except for mean values ${\hat{\varepsilon }}_{k}\sim N(\mu_{k},\sigma_{k}^{2})$ and $q_{k}^{2}\sim \chi^{2}(n_{DOF,k},\mu_{Q,k}^{2})$. Thus, Equation (16) can be upper-bounded using receiver autonomous integrity monitoring (RAIM) methods [6,7,34,35,36,37]. A UO causes a shift in the mean of ${\hat{\varepsilon }}_{k}$ and in the NCP of $q_{k}^{2}$. Large UO-induced feature measurement errors cause large ${\hat{\varepsilon }}_{k}$ (i.e., high risk of HI) but also cause large $q_{k}^{2}$, which makes the UO easier to detect (i.e., low risk of ND).

To analyze this tradeoff, innovation-based chi-squared RAIM methods consider the failure mode slope (FMS) [34,35,36,37]. Given a UO hypothesis $H_{h}$ for h≠0, the FMS is the ratio of the mean estimation error over the NCP of the test statistic $g_{h,k}\equiv {\left(\mu_{k}^{2}/\mu_{Q,k}^{2}\right)}^{1/2}$. Recent analytical results in [35] were established in the context of GNSS/INS integration. They provide the means to recursively determine the FMS when using an EKF for estimation and a sequence of innovations for detection. We use this method to determine the bound in Equation (16) for the risk-maximizing hypothesis $H_{h}$ for $h=0,\ldots ,n_{H}$, i.e., for the worst-case FMS $g_{MAX,k}\equiv \underset{h=1,\ldots ,n_{H}}{\max}g_{h,k}$:

(17) $$P(HI_{k}\cap ND_{k}|CA_{K})\leq \underset{\eta }{\max}\left[\left(Q\left\{\frac{\ell +\eta g_{MAX,k}}{\sigma_{k}}\right\}+Q\left\{\frac{\ell -\eta g_{MAX,k}}{\sigma_{k}}\right\}\right)P_{NC\chi^{2}}\left\{n_{DOF,k},\eta^{2},T_{k}^{2}\right\}\right]$$

where η is a search parameter (called the fault magnitude in [36]) that is easily determined at each time step k using a one-dimensional search, e.g., using an interval-halving method [36], and where

(18) $$P_{NC\chi^{2}}\left(n_{DOF,k},\eta^{2},T_{k}^{2}\right)\equiv \int_{0}^{T_{k}^{2}}\chi_{\tau }^{2}(n_{DOF,k},\eta^{2})d\tau$$

### 4.2. Risk of Incorrect Association Due to Undetected UO

This subsection aims at evaluating the other unknown term in Equation (13): $P(ND_{K}\cap IA_{K})$. The presence of a UO can cause the risk of IA to grow without bound. In this case again, the detector is leveraged to limit the impact of UO on safety risks. However, in contrast with Section 4.1, two major challenges must be tackled to upper-bound $P(ND_{K}\cap IA_{K})$:

(i) the events $IA_{K}$ and $ND_{K}$ are correlated because both events depend on the same innovation vectors; and
(ii) unlike on the left-hand side in Equation (17), there is no condition on association (no “given $CA_{K}$”), so we do not know which association is used to compute the innovations in the detection test statistic $q_{k}^{2}$.

In response, we used an approach based on the minimum detectable error (MDE) concept used in the GPS Local Area Augmentation System (LAAS) [4,38,39]. The MDE is a probabilistic bound on the NCP of the chi-squared detection test statistic. The Appendix A shows that

(19) $$P(ND_{k}\cap IA_{K})\leq \sum_{l=0}^{k}\left(P_{NC\chi^{2}}\left\{n_{l}+m_{l},\mu_{MDE,l}^{2},\frac{L_{l}^{2}\lambda_{l}^{2}}{4}\right\}+I_{MDE,l}\right)$$

where $\mu_{MDE,l}^{2}$ is the MDE due to a UO at time l. $\mu_{MDE,l}^{2}$ can be computed using the following equation:

(20) $$\int_{0}^{T_{l}^{2}}\chi_{\tau }^{2}(n_{l},\mu_{MDE,l}^{2})d\tau =I_{MDE,l}$$

The probability $I_{MDE,l}$ is an integrity risk requirement allocation, i.e., a fraction of $I_{REQ,l}$ such that $I_{MDE,l}<<I_{REQ,l}$. $\mu_{MDE,l}^{2}$ is the smallest value that the detection test statistic NCP can take to ensure that the risk of no detection stays below $I_{MDE,l}$. $\mu_{MDE,l}^{2}$ is a probabilistic bound, not a random variable (which addresses challenge (i) above), and is independent of the association candidate (Equation (20) only depends on the number of degrees of freedom, thus addressing (ii)).

### 4.3. Summary of the New Integrity Risk Bound, Accounting for Presence of UO

In the presence of UOs due to wrong landmark feature extraction, the probability of hazardous misleading information (HMI) at time k can be bounded by the following expression:

(21) $$P(HMI_{k})\leq P(HI_{k}\cap ND_{k}|CA_{K})+P(ND_{k}\cap IA_{K})+I_{FE,k}$$

with

(22) $$P(HI_{k}\cap ND_{k}|CA_{K})\leq \underset{\eta }{\max}\left[\left(Q\left\{\frac{\ell +\eta g_{MAX,k}}{\sigma_{k}}\right\}+Q\left\{\frac{\ell -\eta g_{MAX,k}}{\sigma_{k}}\right\}\right)P_{NC\chi^{2}}\left\{\sum_{l=0}^{k}n_{l},\eta^{2},T_{k}^{2}\right\}\right]$$

(23) $$P(ND_{k}\cap IA_{K})\leq \sum_{l=0}^{k}\left(P_{NC\chi^{2}}\left\{n_{l}+m_{l},\mu_{MDE,l}^{2},\frac{L_{l}^{2}\lambda_{l}^{2}}{4}\right\}+I_{MDE,l}\right)$$

where
$\mu_{MDE,l}^{2}$is derived from $\int_{0}^{T_{k}^{2}}\chi_{\tau }^{2}(n_{l},\mu_{MDE,l}^{2})d\tau =I_{MDE,l}$ and where, in addition to the variables defined under Equations (1)–(3), we used:ηis a scalar search parameter (fault magnitude) that is varied to maximize the integrity risk at each time k;$g_{MAX,k}$is the worst-case failure mode slope (FMS) over all UO hypotheses, determined using the method given in [35];$P_{NC\chi^{2}}\{dof,\mu^{2},T\}$is the probability that a non-centrally chi-squared distributed random variable with “*dof*” degrees of freedom and noncentrality parameter $\mu^{2}$ is lower than some value *T*;$T_{k}^{2}$*.*is a detection threshold set in accordance to a continuity risk requirement $C_{REQ}$ in Equation (11);$I_{MDE,l}$is an integrity risk budget allocation, i.e., a fraction of $I_{REQ,k}$, chosen to satisfy $I_{MDE,k}<<I_{REQ,k}.$

## 5. Performance Analysis

In this section, example simulations and testing introduced in [26,27,28,40,41] are employed to compare the $P(HMI_{k})$ bounds assuming no UOs in Equations (1)–(3) versus accounting for possible UOs in Equations (21)–(23). 

### 5.1. Direct Simulation: Vehicle Roving through a GNSS-Denied Area

This analysis investigated the safety performance of a GPS/LiDAR navigation system onboard a vehicle roving through a forest-type environment. GPS signals were blocked by the tree canopy, and low-elevation satellite signals did not penetrate under the trees. Tree trunks served as landmarks for a two-dimensional LiDAR using a SLAM-type algorithm. 

The measurement vector ${\hat{z}}_{k}$ in Equation (4) was augmented with GPS code and carrier measurements. The state vector $x_{k}$ was augmented to include an unknown GPS receiver clock bias and carrier phase cycle ambiguities. Time-correlated GPS signals and nonlinear LiDAR data were processed in a unified time-differencing EKF derived in [33,34]. The main simulation parameter values are listed in Table 1, and a differential GPS measurement error model was used, which is fully described in [41]. In this scenario, GPS and LiDARs essentially relayed each other with seamless transitions from open sky through GPS-denied areas where landmarks were modeled as poles with nonzero radii. 

**Figure 2:**
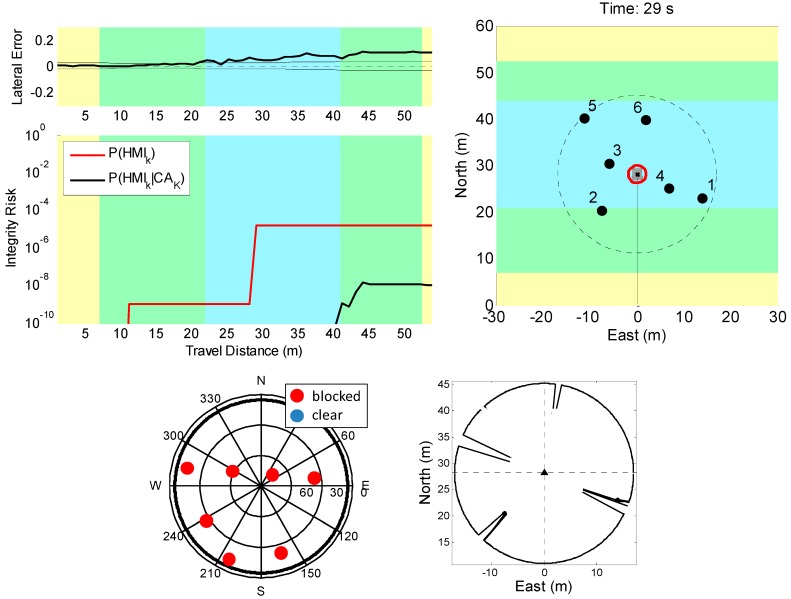
Simulation results assuming no unwanted objects (UO). (**top left**) On the upper plot, the thick black line represents the actual cross-track positioning error and the thin line is the one-sigma covariance envelope. The lower plot shows *P*(*HI_k_*) bounds for the GPS-denied area crossing scenario. (**top right**) Snapshot vehicle-landmark geometry at the time step corresponding to the large increase in *P*(*HI_k_*) Bound (time = 29 s). (**bottom left**) Azimuth elevation sky plot showing GPS satellite geometry at time = 29 s. (**bottom right**) Snapshot LiDAR scan at time = 29 s when landmark “1” is hidden behind landmark “4”.

**Figure 3:**
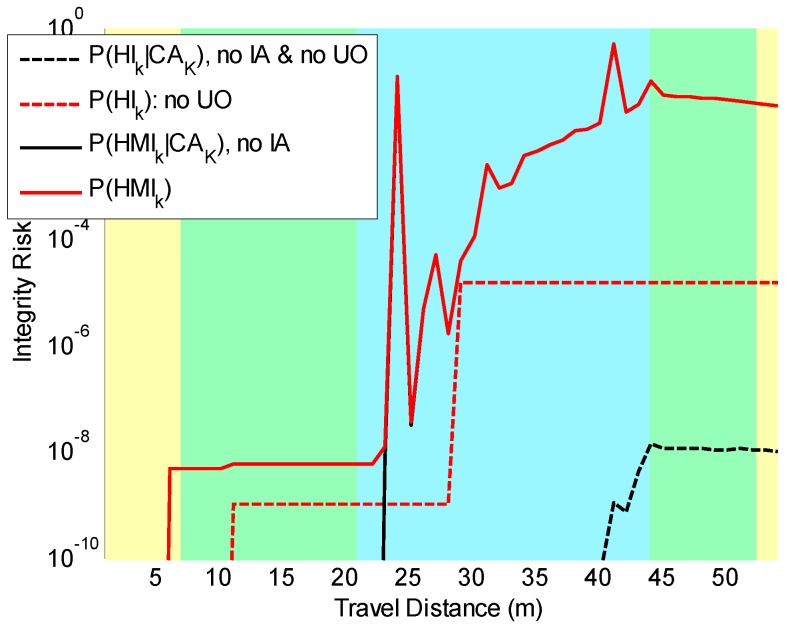
*P*(*HMI_k_*) bounds taking into account the possibility of IA and the potential presence of UOs. The difference between the dashed black line and the solid black line quantifies the impact on *P*(*HMI_k_*) of undetected UOs when assuming correct association (CA). The difference between the dashed red line and the solid red line measures the impact on *P*(*HMI_k_*) of undetected UOs when accounting for incorrect associations.

**Figure 4:**
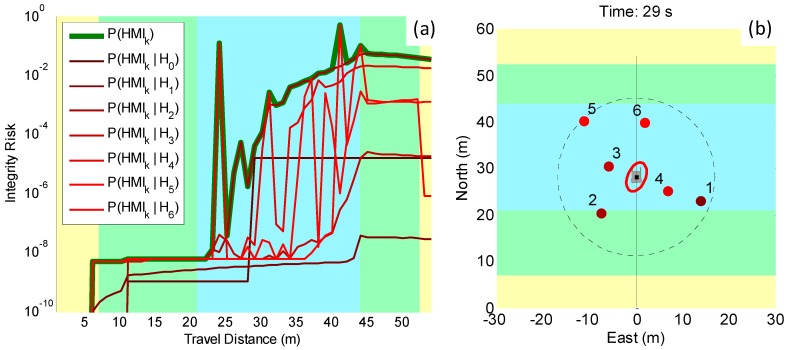
Simulation results accounting for UOs. (**a**) *P*(*HMI_k_*)-bound contributions under each UO hypothesis (*H*_0_ assumes no UO, *H*_1_ assumes a UO masks landmark “1”, etc.): the overall risk is the thick green line. (**b**) Color-coded landmark geometry: the color code identifies which landmark is masked by a UO under the corresponding hypothesis in the left-hand-side plot.

As shown in Figure 2, Figure 3 and Figure 4 and 6, we consistently employed the following yellow-green-blue color code: the mission started with the vehicle operating in a GPS available area (yellow-shaded). Satellite signals available during initialization enabled accurate estimation of cycle ambiguities, so that vehicle positioning uncertainty did not exceed a few centimeters. Then, as the vehicle moved and crossed the GPS- and LiDAR-available area (green-shaded) and the LiDAR-only area (blue-shaded), seamless variations in covariance were achieved. A detailed description of this simulation is given in [41]. In this scenario, the likelihood of IA is high. 

First, as shown in Figure 2, we assumed that no UO was present but IAs occurred. One indicator of IA is displayed on the top of the upper left-hand-side (LHS) plot in Figure 2. It shows that the actual cross-track positioning error (thick black line) versus distance travelled exceeded the corresponding one-sigma covariance envelope (thin black line). This suggests that errors impacting positioning are not captured by the covariance.

This is confirmed on the lower part of the upper LHS chart in Figure 2, where the black curve showing the $P(HI_{k}|CA_{K})$ bound stayed below $10^{-7}$. This curve can directly be derived from the EKF covariance. It does not account for IA. In contrast, the red $P(HI_{k})$-bound curve reached a first plateau of $I_{FE,k}$= 10^−9^ as soon as two landmarks were visible by design of our risk evaluation method [28]. The $P(HI_{k})$ curve then suddenly increased to 10^−5^ at approximately 29 m of travel distance. 

To explain this sudden jump, the top right-hand-side (RHS) chart in Figure 2 shows that, at the travel distance of 29 m (i.e., at travel time = 29 s) corresponding to the large increase in predicted integrity risk, landmark “1” was hidden behind landmark “4”. To the LiDAR, landmark “1” became visible again at the next time step, which made correct measurement association with either landmark “1” or “4” extremely challenging. The $P(HI_{k})$ bound accounted for the risk caused by such events. This is consistent with other results presented in [1,26,27,28].

The bottom LHS chart in Figure 2 shows the simulated GPS satellite geometry on an azimuth elevation plot of the sky. At travel time 29 s, the tree canopy blocked all satellite signals. The bottom RHS chart displays the simulated LiDAR measurements showing again that landmark “1” was not visible from the LiDAR’s viewpoint.

In Figure 3, the risk of having a UO occluding a landmark is taken into account. Our new integrity risk evaluation method was implemented. We could quantify the impact on *P*(*HMI_k_*) of undetected UOs assuming systematic CA by measuring the difference between the dashed black line $P(HI_{k}|CA_{K})$ derived using [28] and the solid black line $P(HMI_{k}|CA_{K})$. We noticed again that $P(HI_{k}|CA_{K})$ (directly derived from the EKF covariance) was a poor safety metric because it stayed below $10^{-7}$, whereas $P(HMI_{k}|CA_{K})$, accounting for UOs, exceeded $10^{-2}$. In parallel, the red curves account for the risk of incorrect association (IA). The difference between the dashed red line and the solid red line, which respectively reached $10^{-5}$ and above $10^{-2}$, shows the impact on *P*(*HMI_k_*) of undetected UOs.

To better understand the shape of the overall $P(HMI_{k})$ bound, Figure 4 shows the contributions of each single-UO hypothesis (assuming no UO, assuming a UO masking landmark “1”, assuming a UO masking landmark “2”, etc.). In Figure 4, the color code used in the LHS graph is also employed in the RHS plot to represent the landmark involved in the corresponding fault hypothesis. Peaks in $P(HMI_{k})$-bound contributions occurred when the landmark geometry and redundancy was too poor to ensure reliable detection of a given UO. The overall $P(HMI_{k})$ bound was the maximum of all the contributions at each time step and is represented with a thick green line.

### 5.2. Preliminary Testing in an Incorrect-Association-Free Environment

**Figure 5:**
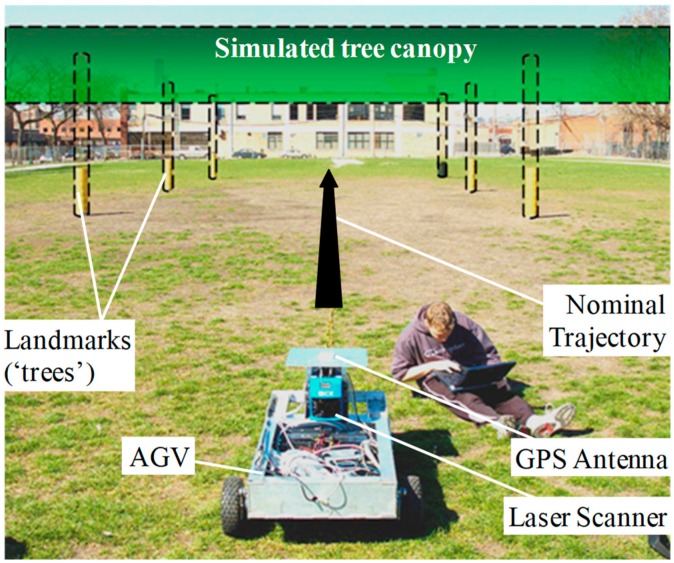
Experimental setup of a forest-type scenario, where a GPS/LiDAR-equipped rover is driving by six landmarks (cardboard columns) in a GPS-denied area. GPS is artificially blocked by a simulated tree canopy and a precise differential GPS solution is used for truth trajectory determination.

Preliminary experimental testing was carried out using data collected in a structured environment shown in Figure 5. Static simple-shaped landmarks were located at locations sparse enough to ensure successful outcomes for FE and DA. Because the results presented here were free of incorrect associations, $P(HMI_{k})$ was expected to match $P(HMI_{k}|CA_{K})$. This test data was used to focus on the risk of UO misdetection.

Measurements from carrier phase differential GPS (CPDGPS) as well as LiDAR scanners were synchronized and recorded. In order to obtain a full 360-degree LiDAR scan, two 180-degree LiDAR scanners were assembled back-to-back. The LiDAR scanners had a specified 15–80-m range limit, a 0.5-degree angular resolution, a 5-Hz update rate, and a ranging accuracy of 1–5 cm (1 sigma) [42]. The GPS antenna was mounted on top of the front LiDAR. The lever-arm distance between the two LiDARs was accounted for. The two LiDARs and the GPS antenna were mounted on a rover also carrying the GPS receiver and data-link. An embedded computer onboard the vehicle recorded all measurements including the raw GPS data from the reference station transmitted via a wireless spread-spectrum data-link. Truth trajectory was obtained using a fixed CPDGPS solution. 

**Figure 6:**
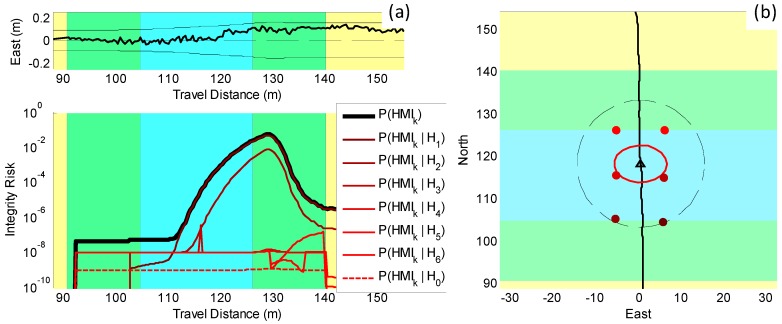
Experimental results accounting for UOs (**a**) *P*(*HMI_k_*)-bound contributions for each unmapped object (UO) hypothesis for the preliminary experimental dataset: the overall risk is the thick black line. (**b**) Color-coded subsets identifying which landmark is occluded by a UO under each one of the six single-UO hypotheses.

The upper LHS chart in Figure 6 confirms that this is an incorrect-association-free scenario because the actual error (thick line) fits within the covariance envelope (thin line) throughout the test. In addition, the lower LHS graph in Figure 6 shows $P(HMI_{k})$-bound contributions for each single-UO hypothesis. The six $P(HMI_{k})$ bounds corresponding to UO hypotheses are shown using the same color code as in Figure 4, and the UO-free hypothesis is the dashed line. The color code is used on the RHS chart, which also shows the landmark geometry. In the LHS graph, $P(HMI_{k})$ increases substantially when accounting for undetected UO (thick black curve), as compared to ignoring their potential presence (dashed red line). UO occluding landmarks “1” and “2” cause by far the largest increase in $P(HMI_{k})$ bound. In this SLAM-type implementation where the map is built incrementally, landmarks observed early in the rover trajectory play a key role throughout the mission, which explains the method’s sensitivity to potential extraction faults on landmarks “1” and “2”. In future work, we will try to reduce the $P(HMI_{k})$ bound using redundant information from other sensors, from additional landmarks, and from additional landmark features.

## 6. Conclusions

This paper presents a new approach to improve the safety of LiDAR-based navigation by quantifying the risks of missed detection of unwanted objects (UO). UOs can occlude useful landmarks, thereby causing large navigation errors. We established a bound on the integrity risk caused by UOs. First, we presented an innovation-based detector, and we established an analytical expression for the impact of undetected UO on the positioning error assuming correct association. Then, we derived a bound on the risk of incorrect association (IA) in the presence of UO. Direct simulation and preliminary testing in a structured environment demonstrated the proposed method’s ability to quantify safety risks in the presence of both UOs and IAs. It showed, for example, that the Kalman filter covariance is a poor metric of safety performance. The analysis of our preliminary experimental results suggests that additional redundant information from other sensors would be needed to safely detect UOs in the LiDAR’s surroundings. 

## Figures and Tables

**Table 1:** Simulation parameters.

System Parameters	Values
Standard deviation of raw LiDAR ranging measurement	0.02 m
Standard deviation of raw LiDAR angular measurement	0.5 deg
LiDAR range limit	20 m
GNSS and LiDAR data sampling interval	0.5 s
Standard deviation of raw GNSS code ranging signal	1 m
Standard deviation of raw GNSS carrier ranging signal	0.015 m
GNSS multipath correlation time constant	90 s
Vehicle speed	1 m/s
Alert limit *ℓ*	0.5 m
Integrity risk allocation for FE, *I_FE,k_*	10^−9^
Integrity risk allocation for MDE, *I_MDE,k_*	10^−10^
Continuity risk requirement, *C_REQ,k_*	10^−3^

## Appendix A. Upper Bound on the Probability of Incorrect Association in the Presence of Unwanted Objects

This appendix aims at finding an upper bound on $P(ND_{K}\cap IA_{K})$. First, we point out that EKF innovations are used both for data association (DA) and for UO detection. Because the sequence of innovations is white, events $ND_{K}$ and $IA_{l}$ are independent for l=0,…,k−1, but not for l=k. Also, it is worth noting that $IA_{K}$ is a union of events of incorrect associations at any previous time steps: $IA_{K}\equiv IA_{k}\cup IA_{k-1}\cup \ldots \cup IA_{0}$, and that by definition of the association initialization [31], $P(IA_{0})=0$. We use these observations to rewrite $P(ND_{K}\cap IA_{K})$ as

(A1) $$\begin{array}{l} P(ND_{K}\cap IA_{K})=P\left(\overset{k}{\underset{l=0}{\cup }}\left(ND_{l}\cap IA_{l}\right)\right)\\ \;=\sum_{l=0}^{k}P(ND_{l}\cap IA_{l})-P\left(\overset{k}{\underset{l=0}{\cap }}\left(ND_{l}\cap IA_{l}\right)\right)\\ \;=\sum_{l=0}^{k}P(ND_{l}\cap IA_{l})-\prod_{l=1}^{k}P(ND_{l}\cap IA_{l}|ND_{L-1}\cap IA_{L-1})P(ND_{0}\cap IA_{0})\\ \;=\sum_{l=0}^{k}P(ND_{l}\cap IA_{l}) \end{array}$$

This expression is desirable because it can be updated recursively. We will upper-bound $P(ND_{K}\cap IA_{K})$ by bounding each individual term in the sum in Equation (A1).

From the definition of the detection test in Section 3.1, the $ND_{l}$ event can be expressed as

$$ND_{l}\equiv \left\{\sum_{j=0}^{l}\underset{i=1,\ldots ,n_{A}}{\min}\gamma_{i,j}^{2}\leq T_{l}^{2}\right\}$$

which is included in the event:

(A2) $$ND_{l*}\equiv \left\{\gamma_{MIN,l}^{2}\leq T_{l}^{2}\right\}\;\mathrm{where}\;\gamma_{MIN,l}^{2}\equiv \underset{i=1,\ldots ,n_{A}}{\min}\gamma_{i,l}^{2}$$

With the index notation “MIN” defined in Equation (A2), the distribution of $\gamma_{MIN,l}^{2}$ is known ($\gamma_{MIN,l}^{2}\sim \chi^{2}(n_{l},y_{MIN,l}^{2})$) except for its NCP $y_{MIN,l}^{2}$. With the knowledge that no detection occurred, we can determine a probabilistic bound $\mu_{MDE,l}^{2}$ on $y_{MIN,l}^{2}$. The law of total probability is used again to express a bound on $P(ND_{K}\cap IA_{K})$ as

(A3) $$\begin{array}{cc} P(ND_{l}\cap IA_{l}) & \leq P(ND_{l*}\cap IA_{l})\\ & \leq P(ND_{l*}\cap IA_{l}\cap y_{MIN,l}^{2}<\mu_{MDE,l}^{2})+P(ND_{l*}\cap IA_{l}\cap y_{MIN,l}^{2}\geq \mu_{MDE,l}^{2})\\ & \leq P(IA_{l}|y_{MIN,l}^{2}<\mu_{MDE,l}^{2})+P(ND_{l*}\cap y_{MIN,l}^{2}\geq \mu_{MDE,l}^{2}) \end{array}$$

We find $\mu_{MDE,l}^{2}$ to ensure that the second term in Equation (A3) is smaller than a predefined allocation $I_{MDE,l}$:

(A4) $$P(\gamma_{MIN,l}^{2}\leq T_{l}^{2}\cap y_{MIN,l}^{2}\geq \mu_{MDE,l}^{2})\leq I_{MDE,l}$$

A minimum value for $\mu_{MDE,l}^{2}$ is found using the expression

(A5) $$P(\gamma_{MIN,l}^{2}\leq T_{l}^{2}|y_{MIN,l}^{2}=\mu_{MDE,l}^{2})=I_{MDE,l}$$

Hence, Equation (20). $\mu_{MDE,l}^{2}$ is the smallest value of the test statistic NCP that can cause no detection with probability lower than $I_{MDE,l}$. Any error larger than that will be detected with probability larger than 1-$I_{MDE,l}$, which is considered safe. Substituting Equation (A4) into (A3), Equation (A3) becomes

(A6) $$P(ND_{l}\cap IA_{l})\leq P(IA_{l}|y_{MIN,l}^{2}<\mu_{MDE,l}^{2})+I_{MDE,l}$$

As described in Section 2, the IA event may be expressed as $IA_{l}\equiv \gamma_{MIN,l}^{2}<\gamma_{0,l}^{2}$ when the “MIN” differs from 0. We address the fact that the random variables $\gamma_{MIN,l}^{2}$ and $\gamma_{0,l}^{2}$ are correlated using the exact same steps as in [28]. The derivation in [28] shows that the following event includes $IA_{l}$:

(A7) $$y_{MIN,l}^{T}Y_{MIN,l}^{-1}y_{MIN,l}\leq 4\;q_{l}^{T}q_{l}$$

where

$y_{MIN,l}$	is defined in Equation (7) and is not zero because of IA (not due to UOs);
$Y_{MIN,l}$	is defined in Equation (9);
$q_{l}$	is an $(n_{l}+m_{l})\times 1$ vector such that $q_{l}\sim N(\mu_{Q,l},I_{n_{l}+m_{l}})$;
**4**	the factor four is derived in [28] by solving an eigenvalue problem involving a sum of two idempotent matrices.

In this work, we distinguish the impacts of the IA and UO. Recall that the CA is the one where all landmarks that are not occluded by a UO are correctly associated, i.e., where the innovation vector would be zero mean if the UO was removed. The mean contribution due to IA is accounted for with $y_{MIN,l}$ on the left-hand side of (A7). In contrast with [28], $q_{l}$ is not zero mean because of the presence of a UO. Following the eigenvalue solution provided in [28], the maximum impact of UO on the right-hand-side term is $4\mu_{MDE,l}^{2}$. After dividing both sides of Equation (A7) by 4, the probability of occurrence of the event in Equation (A7) is expressed in Equation (19).
